# The Impact of Vitamin D Supplementation Duration on Early Childhood Developmental Milestones: A Retrospective Study

**DOI:** 10.3390/nu16244395

**Published:** 2024-12-20

**Authors:** Andrea D. Praticò, Manuela Lo Bianco, Roberta Leonardi, Agata Polizzi, Martino Ruggieri

**Affiliations:** 1 Unit of Pediatrics, Department of Medicine and Surgery, University Kore of Enna, 94100 Enna, Italy; 2 Unit of Pediatric Clinic, Department of Clinical and Experimental Medicine, University of Catania, 95123 Catania, Italy; 3 Postgraduate Residency Program in Pediatrics, University of Catania, 95123 Catania, Italy; leonardi.roberta@outlook.it

**Keywords:** vitamin D, neurodevelopment, treatment, prophylaxis

## Abstract

Background: Vitamin D plays a pivotal role in early childhood development, influencing skeletal strength, neuromuscular coordination, and neurodevelopment. This study aimed to evaluate the impact of different durations of Vitamin D supplementation on achieving developmental milestones. Methods: A retrospective study was conducted on 209 children, divided into two cohorts based on Vitamin D supplementation duration: six months (*n* = 102) and twelve months (*n* = 107). Developmental milestones were assessed across motor (sitting, walking), fine motor (object tracking, grasping), and social (smiling, speech) domains. Statistical analyses, including *t*-tests and effect size calculations, were performed to compare the mean ages of milestone achievement. Results: The 12-month group achieved several milestones significantly earlier than the 6-month group. These included walking, object tracking, and combining words into phrases. Differences in other milestones, such as sitting and smiling, were not statistically significant. Effect sizes ranged from small to moderate. Conclusion: Extended Vitamin D supplementation is associated with modest yet significant advancements in key developmental milestones. However, socio-environmental factors, including parental involvement, likely contributed to these differences. This study’s retrospective design limits causal interpretation, emphasizing the need for prospective, randomized studies to validate findings. These results support the potential benefits of extending Vitamin D supplementation beyond six months to optimize developmental outcomes in infants.

## 1. Introduction

Vitamin D, commonly known as the “sunshine vitamin”, is a fat-soluble nutrient that plays a pivotal role in maintaining calcium homeostasis and promoting bone mineralization. Historically, its deficiency has been linked to conditions like rickets in children and osteomalacia in adults, highlighting its importance in skeletal health [1].

However, emerging research over the past two decades has expanded the understanding of Vitamin D’s role, demonstrating its critical involvement in extra-skeletal systems, including the immune system, cardiovascular regulation, and neurodevelopment [2].

The first year of life is a dynamic period of rapid growth and brain development. During this time, processes such as neuronal differentiation, synaptogenesis, and myelination are at their peak, laying the foundation for motor skills, cognitive functions, and social behaviors. Adequate nutrition during infancy is vital to support these processes, with Vitamin D increasingly recognized as an essential nutrient for neurodevelopmental health. This is due, in part, to the widespread distribution of Vitamin D receptors (VDRs) in the brain, particularly in regions such as the hippocampus, cerebellum, and prefrontal cortex, which are responsible for motor control and cognitive development [3]. Through these receptors, Vitamin D regulates key processes, including calcium signaling, neuroprotection, and the production of neurotrophic factors essential for brain development [4,5].

Developmental milestones serve as critical benchmarks for assessing the growth and maturation of infants and young children. These milestones encompass physical, cognitive, and social abilities, reflecting the integrated development of various biological systems. Motor milestones, such as sitting, standing, and walking, depend on skeletal strength and neuromuscular coordination. Fine motor milestones, like grasping and tracking objects, require precise coordination of the central and peripheral nervous systems. Social and cognitive milestones, including smiling, eye contact, and speech, indicate the development of early social and linguistic skills. Tracking the achievement of these milestones provides valuable insights into a child’s overall neurodevelopment and can highlight potential delays or deficiencies.

Vitamin D is thought to influence these milestones through its multifaceted roles in skeletal and neural development. By regulating calcium metabolism, it contributes to bone strength and neuromuscular coordination, directly impacting physical abilities like standing and walking [6]. Beyond these skeletal effects, Vitamin D has neurotrophic properties, supporting synaptogenesis, myelination, and overall brain health. Deficiency in Vitamin D has been associated with delays in motor and cognitive milestones, further underscoring its significance [7]. However, while the role of Vitamin D in supporting neurodevelopment is well documented, questions remain about the optimal duration and dosing of supplementation during infancy.

Current guidelines recommend daily supplementation of 400 IU of Vitamin D for all infants, particularly in regions with limited sunlight exposure [6]. While this recommendation is generally adhered to during the first six months of life, extending supplementation beyond this period is less consistently practiced, with limited empirical evidence to support its additional benefits. The potential advantages of prolonged supplementation for achieving developmental milestones remain an area of active research.

This study aims to address this knowledge gap by comparing the developmental outcomes of two cohorts of children who received Vitamin D supplementation for different durations during their first year of life. Specifically, the study examines motor, fine motor, and social milestones in children supplemented for six months versus twelve months, with the goal of determining whether extended supplementation confers measurable developmental advantages. By comparing observed milestone achievement against established population norms, the study provides valuable insights into the impact of Vitamin D on early childhood development and its potential role in optimizing long-term neurodevelopmental trajectories.

The findings of this research contribute to the growing body of literature advocating for evidence-based nutritional interventions during infancy. Previous studies, such as those by Holick [1] and Wagner and Greer [6], have established Vitamin D’s role in skeletal and neuromuscular health, while more recent work by Camargo et al. [7] and Carlberg and Haq [4] has highlighted its neuroprotective effects. By focusing on the duration of supplementation, this study addresses a specific gap in the literature, providing actionable insights for clinicians and caregivers seeking to optimize infant health through early-life nutrition.

## 2. Patients and Methods

This study included a total of 209 children divided into two cohorts based on the duration of Vitamin D supplementation during their first year of life. The first cohort consisted of 102 children (49 males and 53 females) who were supplemented with 400 IU of Vitamin D daily for six months. The second cohort included 107 children (52 males and 55 females) who received the same daily dose of Vitamin D for twelve months. The mean age of the children at the time of assessment was 3.1 years (±0.3) in the six-month group and 3.2 years (±0.3) in the twelve-month group. Socio-economic status was comparable in the two groups (level of education of the parents: Master’s Degree or above in 21% (6-month group) and 23% (12-month group); secondary in 56 and 55%, respectively; primary or lower in 23% and 22%, respectively). The study was conducted on a sample pediatric population coming from the Eastern Provinces of Sicily (Catania, Siracusa, Enna, Ragusa) in the Unit of Pediatric Clinic and Unit of Rare Disorders of the Nervous System in Childhood, University of Catania, Italy, and in the Unit of Pediatrics of the University Kore of Enna. The study was approved by the Ethical Committee of the University of Enna (253/2023). Written informed consent for publication was obtained from participating parents of the patients.

All participants were recruited from pediatric and neonatology clinics where routine supplementation was implemented as part of standard care. Inclusion criteria were as follows: healthy-term infants born without any known genetic or metabolic disorders, no history of major perinatal complications, and no significant medical conditions that could interfere with developmental outcomes. 

The developmental milestones assessed in this study were grouped into three domains: motor milestones (sitting, standing, and walking), fine motor milestones (grasping objects and object tracking), and social milestones (smiling, eye contact, first words, and speech). These milestones were chosen based on their importance in reflecting the integrated development of musculoskeletal, neurological, and cognitive systems during early childhood. The milestones were recorded through a combination of caregiver interviews and direct pediatric observation at routine check-ups. Additionally, the Griffiths Developmental Scale was utilized to assess global developmental performance at 1 and 2 years of age, providing a comprehensive evaluation of neurodevelopmental outcomes. Lastly, Vitamin D levels were assessed at 6 and 12 months, respectively, in all the patients.

## 3. Statistical Analysis

Data were analyzed using descriptive and inferential statistical methods. Continuous variables, such as the age of milestone achievement, were summarized as means with standard deviations (SDs). Categorical variables, such as gender distribution, were presented as counts and percentages.

Comparisons between the two groups were conducted using independent-sample *t*-tests for continuous variables to evaluate differences in the mean ages of milestone achievement. For categorical variables, chi-square tests were employed to examine differences in gender distribution. Statistical significance was set at a two-tailed *p*-value of <0.05.

The primary outcomes of interest were the mean ages at which developmental milestones were achieved in each cohort. To determine whether the observed differences between the two groups were statistically significant, 95% confidence intervals (CIs) were calculated for mean differences in milestone ages. Additionally, effect sizes (Cohen’s d) were calculated for significant findings to evaluate the magnitude of the differences.

Secondary analyses included a comparison of the observed mean ages for each milestone in both groups against established population norms. This was performed using one-sample *t*-tests, with deviations from normative values reported as statistically significant if the *p*-value was <0.05.

All statistical analyses were performed using SPSS (version 26.0, IBM Corp., Armonk, NY, USA) and GraphPad Prism (version 9.0). The robustness of the findings was further assessed through sensitivity analyses, which excluded outliers defined as data points falling more than 2.5 SD from the group mean.

## 4. Results

### 4.1. Baseline Characteristics

A total of 209 children were included in the study, divided into two cohorts based on the duration of Vitamin D supplementation. The first cohort (6-month group) consisted of 102 children, with a gender distribution of 49 males (48%) and 53 females (52%) and a mean age at assessment of 3.1 years (±0.3). The second cohort (12-month group) comprised 107 children, with 52 males (49%) and 55 females (51%), and a mean age at assessment of 3.2 years (±0.3). Both groups were comparable in terms of baseline demographic characteristics, with no statistically significant differences in age or gender distribution (*p* > 0.05).

### 4.2. Developmental Milestone Achievement

The mean ages at which key motor, fine motor, and social milestones were achieved are detailed below. Data are presented as mean ages (±SD), with comparisons between the two groups (Table 1).

### 4.3. Motor Milestones

Sitting: The mean age for achieving independent sitting was 6.10 months (±0.15) in the 6-month group and 6.07 months (±0.12) in the 12-month group, with a non-significant difference (*p* = 0.113).

Standing: Children in the 6-month group stood independently at a mean age of 9.80 months (±0.20) compared to 9.76 months (±0.18) in the 12-month group, with no significant difference (*p* = 0.131).

Walking: Walking unassisted was achieved at a mean age of 12.30 months (±0.25) in the 6-month group and 12.10 months (±0.22) in the 12-month group. The difference was statistically significant (*p* < 0.01).

Fine Motor Milestones

Grasping objects: The mean age for achieving grasping of objects was 5.90 months (±0.14) in the 6-month group and 5.87 months (±0.10) in the 12-month group, with a non-significant difference (*p* = 0.078).

Object Tracking: The ability to track moving objects was achieved at 10.06 weeks (±0.20) in the 6-month group compared to 9.98 weeks (±0.18) in the 12-month group. The difference was statistically significant (*p* < 0.01).

### 4.4. Social Milestones

Smiling: The first social smile was observed at 6.50 weeks (±0.20) in the 6-month group and 6.48 weeks (±0.18) in the 12-month group, with no significant difference (*p* = 0.75).

Visual Contact: The mean age for establishing sustained visual contact was 8.20 weeks (±0.15) in the 6-month group and 8.17 weeks (±0.12) in the 12-month group, with no significant difference (*p* = 0.113).

First Words: The mean age at which children spoke their first words was 13.40 months (±0.30) in the 6-month group and 13.26 months (±0.25) in the 12-month group. The difference approached significance (*p* < 0.01).

Speech (Phrases): Combining words into phrases was achieved at 24.10 months (±0.35) in the 6-month group compared to 23.97 months (±0.30) in the 12-month group. This difference was statistically significant (*p* < 0.01).

Griffiths Score: At both 1 and 2 years of age, Griffiths Scores were significantly higher in the 12-month Vitamin D supplementation group compared to the 6-month group (*p* < 0.001 and *p* = 0.018, respectively). This suggests that extended supplementation is associated with modest but meaningful improvements in developmental outcomes.

Vitamin D levels were not statistically different at 6 months of age, while at 12 months of age a statistically significant difference was observed (see Table 2 and Table 3).

### 4.5. Comparison with Population Averages

Both groups achieved developmental milestones within the expected population averages. However, the 12-month group demonstrated a closer alignment with earlier normative thresholds for several milestones, including walking, object tracking, and speech. No significant delays were observed in either cohort relative to population norms.

### 4.6. Effect Sizes

The effect sizes (Cohen’s d) for significant differences between groups were small to moderate, ranging from 0.20 to 0.35, indicating that the observed differences, while statistically significant, were of modest practical magnitude.

### 4.7. Sensitivity Analysis

Excluding outliers (values > 2.5 SD from the mean) did not materially alter the findings, supporting the robustness of the results.

## 5. Discussion

This study aimed to assess the impact of Vitamin D supplementation duration on the achievement of early childhood developmental milestones. By comparing two cohorts of children, one receiving supplementation for six months and the other for twelve months, we identified statistically significant differences in several key milestones, particularly in motor and fine motor domains. While these differences were modest in magnitude, they highlight the potential benefits of extending Vitamin D supplementation beyond the first six months of life.

### 5.1. Comparison Between the Groups

The 12-month supplementation group demonstrated statistically significant advancements in several developmental milestones compared to the 6-month group. Notably, walking, object tracking, and speech (phrases) were achieved at earlier ages in the 12-month group, with effect sizes indicating moderate practical significance. These findings align with existing evidence suggesting that Vitamin D plays a critical role in skeletal, neuromuscular, and neurocognitive development [1,2].

For motor milestones, earlier achievement of walking in the 12-month group underscores the importance of adequate Vitamin D levels for neuromuscular coordination and skeletal strength [3]. Similarly, fine motor milestones such as object tracking were slightly accelerated in the 12-month group, possibly reflecting enhanced neural processing and coordination, consistent with the neurotrophic effects of Vitamin D [4]. Speech development, including the combination of words into phrases, was also achieved earlier in the 12-month group, suggesting potential cognitive and language-related benefits [5,6].

### 5.2. Potential Confounding Factors

An important consideration in interpreting these findings is the potential influence of socio-environmental factors. Families who adhered to the 12-month supplementation regimen may represent a subset of caregivers who were more proactive in their child-rearing practices [7]. Enhanced parental involvement, increased stimulation, and greater access to resources could have independently contributed to the observed differences in developmental milestones [8]. Previous research has shown that enriched environments during early childhood significantly enhance neurodevelopment, independent of nutritional factors [9,10].

While our results suggest a beneficial role of extended Vitamin D supplementation, the possibility that these differences partially reflect variations in familial or environmental factors cannot be excluded. This highlights the need for future studies to control for such confounding variables, ensuring that the effects of supplementation are isolated from other influences. This can also be underlined by the fact that several statistically differences were seen in neurodevelopmental milestones reached before the end of Vitamin D treatment (object tracking)

### 5.3. Biological Mechanisms

The observed advancements in developmental milestones among the 12-month group are consistent with the established biological roles of Vitamin D in early development. Vitamin D regulates calcium homeostasis, which is essential for skeletal mineralization and neuromuscular function [11]. Beyond its skeletal effects, Vitamin D receptors are expressed in the brain, where they modulate neurotrophic factors, neuronal differentiation, and synaptic plasticity [4,12]. These mechanisms likely underpin the earlier achievement of motor and fine motor milestones in the 12-month group.

Furthermore, studies have demonstrated that Vitamin D deficiency during infancy is associated with delayed cognitive and motor development [6]. Prolonged supplementation may mitigate such risks, supporting optimal neurodevelopment during critical early-life windows [13].

### 5.4. Study Limitations

This study has several limitations that must be considered. First, the retrospective design inherently limits the ability to infer causality. While our findings are suggestive of a positive association between longer supplementation and earlier milestone achievement, prospective studies are required to confirm these results [14].

Second, the absence of randomization introduces potential selection bias. Families in the 12-month group may differ systematically from those in the 6-month group, not only in adherence to supplementation but also in socio-economic status, education, and caregiving practices, all of which are known to influence child development [9,10]. Future studies should collect and adjust for such variables to better isolate the effects of Vitamin D.

Third, this study relied on caregiver reports and pediatric assessments, which, while valuable, are subject to recall and reporting bias. Objective measures of development, such as standardized tests, would strengthen the validity of the findings [15].

Finally, the fixed dose of 400 IU of Vitamin D was used for all participants. While this dose aligns with current guidelines [6], higher doses may yield additional benefits, as suggested by emerging evidence [8,16]. Future studies should explore dose–response relationships to optimize recommendations.

### 5.5. Future Directions

To address these limitations, prospective studies designed in a randomized, double-blind manner are needed. Such studies would allow for more rigorous control of confounding factors, enabling a clearer understanding of the causal relationship between Vitamin D supplementation duration and developmental outcomes [17]. Moreover, longitudinal designs could assess the long-term impacts of supplementation on academic performance, social functioning, and mental health, areas increasingly recognized as influenced by early-life nutrition [7,18].

## 6. Conclusions

This study contributes to the growing body of evidence supporting the importance of Vitamin D in early childhood development. While both six-month and twelve-month supplementation effectively supported milestone achievement within normative ranges, the 12-month group demonstrated modest but significant advantages in some (but not all) motor, fine motor, and social domains. These findings highlight the potential benefits of extending Vitamin D supplementation beyond the currently recommended duration, particularly in populations at risk of deficiency.

However, the influence of socio-environmental factors underscores the complexity of interpreting these results. Careful consideration of confounding variables, combined with rigorous prospective studies, will be critical in refining supplementation guidelines and maximizing developmental outcomes in children [19,20].

## Figures and Tables

**Table 1:** Motor, fine motor, and social milestones for 102 children treated with 400 IU Vitamin D for 6 months and 107 patients treated for 1 year.

Milestone	6-Month Vitamin D Group (102 Children)	12-Months Vitamin D Group (107 Children)
Sitting (months)	6.10 (±0.15)	6.07 (± 0.12)
Object grasping (months)	5.90 (±0.14)	5.87 (± 0.10)
Standing (months)	9.80 (± 0.20)	9.76 (±0.18)
Walking (months)	12.30 (±0.25)	12.10 (± 0.22) *
Smiling (weeks)	6.50 (±0.20)	6.48 (± 0.18)
First Words (months)	13.40 (±0.30)	13.26 (±0.25)
Speech (months)	24.10 (±0.35)	23.97 (± 0.30) *
Visual Contact (weeks)	8.20 (± 0.15)	8.17 (± 0.12)
Following objects (weeks)	10.06 (± 0.20)	9.98 (± 0.12) *
Griffiths Score at 1 year	90.5 (±5.2)	92.7 (±4.9)
Griffiths Score at 2 year	98.3 (±6.0)	100.1 (±5.7)

* Difference is statistically significant (*p* < 0.05).

**Table 2:** Serum levels of Vitamin D at 6 months of age in the two groups.

Group	Number of Infants (n)	Mean Serum Vitamin D Level at 6 Months (ng/mL)	Standard Deviation (±SD)	Optimal Range (20–50 ng/mL)
6-Month Supplementation	102	33.4	5.5	95%
12-Month Supplementation	107	33.2	6.1	94%

**Table 3:** Serum levels of Vitamin D at 12 months of age in the two groups.

Group	Number of Infants (n)	Mean Serum Vitamin D Level at 12 Months (ng/mL)	Standard Deviation (±SD)	Optimal Range (20–50 ng/mL)
6-Month Supplementation	102	27.4	6.5	82%
12-Month Supplementation	107	34.2	7.3	92%

## Data Availability

The original contributions presented in this study are included in the article. Further inquiries can be directed to the corresponding author.
